# Supplementary material for: Anticandidal Activity of Capsaicin and Its Effect on Ergosterol Biosynthesis and Membrane Integrity of Candida albicans
Source: Int J Mol Sci. 2023 Jan 5;24(2):1046. doi: 10.3390/ijms24021046 (PMC9860720; doi:10.3390/ijms24021046)
Supplement: Supplementary file 1 [file ijms-24-01046-s001.zip › ijms-2021452-supplementary.pdf]

Supplementary Figure S1. The (1a,2a,3a) represents the CLSM image of the mature biofilm of C. albicans, whereas the (1b,2b,3b) represents the biofilm morphology after the treatment

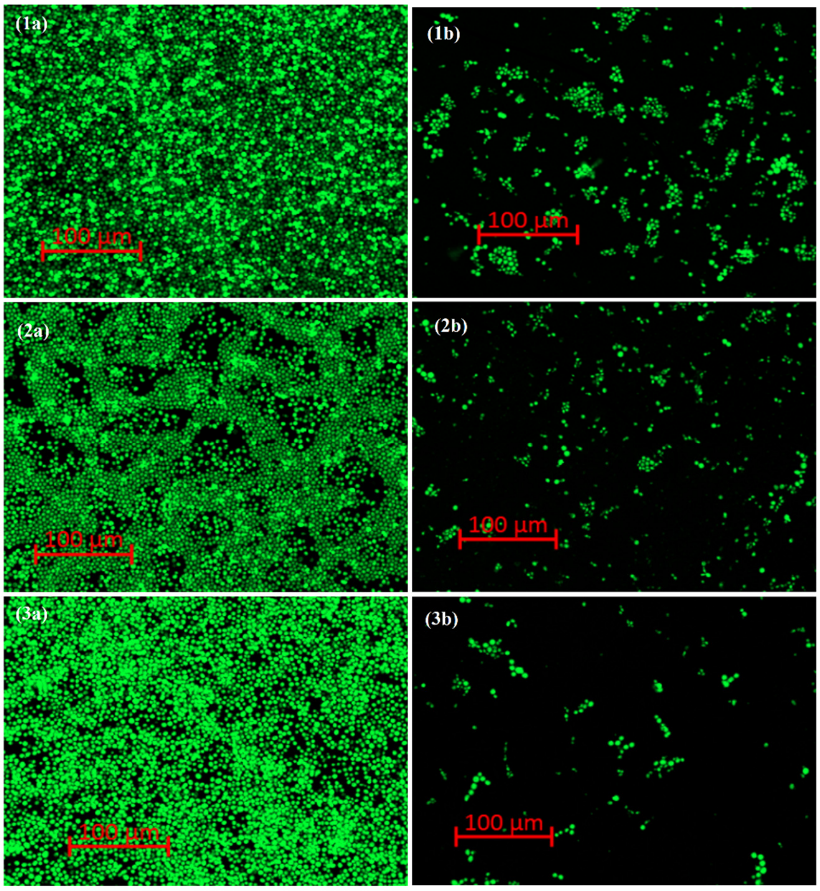

Supplementary Table S1. *Synersgistic effect of Capsiacin (CAP) and fluconazol (FLC) agaist the oral and endodontic isolates (Endo) and standard ATCC strain. Experiment was performed in triplicates.*

Species name & code		MIC (µg/mL)						
		CAP (Alone)	CAP (Combo)	CAP (FICI)	FLC (Alone)	FLC (Combo)	FLC (FICI)	Σ FICI
Reference strains	*C. glabrata* ATCC 15126	12.5	6.25	0.500	16	8	0.500	1.000
	C. albicans ATCC 24433	25	6.25	0.250	64	4	0.063	0.313
Oral isolates	*C. albicans* ORS 1	12.5	3.125	0.250	32	4	0.125	0.375
	C. dubliniensis ORS 2	25	6.25	0.250	32	2	0.063	0.313
	C. albicans ORS 3	12.5	6.25	0.500	64	16	0.250	0.750
	C. albicans ORS 4	25	12.5	0.500	128	16	0.125	0.625
	C. dubliniensis ORS 5	12.5	3.125	0.250	8	2	0.250	0.500
	C. lusitaniae ORS 6	25	12.5	0.500	4	2	0.500	1.000
	C. tropicalis ORS 7	12.5	3.125	0.250	16	4	0.250	0.500
	C. albicans ORS 8	25	12.5	0.500	8	4	0.500	1.000
	C. tropicalis ORS 9	25	6.25	0.250	64	16	0.250	0.500
	C. dubliniensis ORS 10	50	6.25	0.125	4	2	0.500	0.625
	C. albicans ORS 11	12.5	3.125	0.250	128	8	0.063	0.313
	C. tropicalis ORS 12	12.5	6.25	0.500	128	16	0.125	0.625
	C. albicans ORS 13	12.5	3.125	0.250	128	8	0.063	0.313
	C. glabrata ORS 14	25	6.25	0.250	128	16	0.125	0.375
	C. albicans ORS 15	50	12.5	0.250	64	8	0.125	0.375
	C. albicans ORS 16	25	12.5	0.500	128	16	0.125	0.625
	C. albicans ORS 17	12.5	6.25	0.500	8	4	0.500	1.000
	C. albicans ORS 18	12.5	3.125	0.250	8	2	0.250	0.500
	C. albicans ORS 19	50	12.5	0.250	16	4	0.250	0.500
	C. albicans ORS 20	25	3.125	0.125	16	4	0.250	0.375
	C. dubliniensis ORS 21	12.5	6.25	0.500	8	4	0.500	1.000
	C. parapsilosis ORS 22	25	6.25	0.250	4	2	0.500	0.750
	C. dubliniensis ORS 23	12.5	3.125	0.250	4	2	0.500	0.750
	C. kefyr ORS 24	25	6.25	0.250	8	2	0.250	0.500
	C. albicans ORS 25	12.5	6.25	0.500	64	8	0.125	0.625
	C. albicans ORS 26	50	12.5	0.250	4	2	0.500	0.750
	C. glabrata ORS 27	50	6.25	0.125	16	8	0.500	0.625
	C. glabrata ORS 28	50	12.5	0.250	64	4	0.063	0.313
	C. albicans ORS 29	50	12.5	0.250	32	4	0.125	0.375
	C. albicans ORS 30	25	6.25	0.250	32	8	0.250	0.500
Endodontic isolates (*C. albicans*)	Endo-902	25	6.25	0.250	64	16	0.250	0.500
	Endo-903	25	6.25	0.250	64	8	0.125	0.375
	Endo-904	25	12.5	0.500	128	16	0.125	0.625
	Endo-905	25	6.25	0.250	64	4	0.063	0.313
	Endo-906	25	6.25	0.250	128	16	0.125	0.375
	Endo-908	25	6.25	0.250	32	8	0.250	0.500
	Endo-910	25	12.5	0.500	16	8	0.500	1.000
	Endo-911	25	12.5	0.500	128	16	0.125	0.625

∑FICI (fractional inhibitory concentration index) ≤ 0.5 indicates synergetic interaction, and >0.5 to 4 indicates no interaction between the two agents. Experiment was performed in triplicates.
